# Obstetric care navigation: a new approach to promote respectful maternity care and overcome barriers to safe motherhood

**DOI:** 10.1186/s12978-017-0410-6

**Published:** 2017-11-13

**Authors:** Kirsten Austad, Anita Chary, Boris Martinez, Michel Juarez, Yolanda Juarez Martin, Enma Coyote Ixen, Peter Rohloff

**Affiliations:** 1 Wuqu’ Kawoq | Maya Health Alliance 2 Calle 5-43, Zona 1, Santiago Sacatepéquez, Guatemala; 2 Division of Women’s Health, Department of Medicine, Brigham and Women’s Hospital, 75 Francis Street, Boston, MA 02115 USA; 3 Department of Emergency Medicine, Massachusetts General Hospital, 55 Fruit Street, Boston, MA 02114 USA; 4 Division of Global Health Equity, Brigham and Women’s Hospital, 75 Francis Street, Boston, MA 02115 USA

**Keywords:** Maternal health, Respectful maternity care, Disrespect and abuse, Maternal mortality, Guatemala, Indigenous health, Quality improvement

## Abstract

**Background:**

Disrespectful and abusive maternity care is a common and pervasive problem that disproportionately impacts marginalized women. By making mothers less likely to agree to facility-based delivery, it contributes to the unacceptably high rates of maternal mortality in low- and middle-income countries. Few programmatic approaches have been proposed to address disrespectful and abusive maternity care.

**Obstetric care navigation:**

Care navigation was pioneered by the field of oncology to improve health outcomes of vulnerable populations and promote patient autonomy by providing linkages across a fragmented care continuum. Here we describe the novel application of the care navigation model to emergency obstetric referrals to hospitals for complicated home births in rural Guatemala. Care navigators offer women accompaniment and labor support intended to improve the care experience—for both patients and providers—and to decrease opposition to hospital-level obstetric care. Specific roles include deflecting mistreatment from hospital staff, improving provider communication through language and cultural interpretation, advocating for patients’ right to informed consent, and protecting patients' dignity during the birthing process. Care navigators are specifically chosen and trained to gain the trust and respect of patients, traditional midwives, and biomedical providers. We describe an ongoing obstetric care navigator pilot program employing rapid-cycle quality improvement methods to quickly identify implementation successes and failures. This approach empowers frontline health workers to problem solve in real time and ensures the program is highly adaptable to local needs.

**Conclusion:**

Care navigation is a promising strategy to overcome the “humanistic barrier” to hospital delivery by mitigating disrespectful and abusive care. It offers a demand-side approach to undignified obstetric care that empowers the communities most impacted by the problem to lead the response. Results from an ongoing pilot program of obstetric care navigation will provide valuable feedback from patients on the impact of this approach and implementation lessons to facilitate replication in other settings.

## Introduction

While maternal mortality has declined by 44% globally from 1990 to 2015, disparities persist [1]. Poor and otherwise marginalized women continue to face the highest risk of death from largely preventable complications of pregnancy and childbirth. Promoting facility-based delivery with a skilled birth attendant for all women is a key strategy to reduce this disparity [2, 3]. Large-scale global interventions to date have included educating mothers and other stakeholders regarding the benefits of facility-based delivery and decreasing transportation barriers. Despite these efforts, over half of women worldwide still deliver at home with an unskilled birth attendant [1], a fact that signals the need to understand and address persistent barriers that women face.

A strong body of literature demonstrates that dissatisfaction with maternity care is an important deterrent to facility-based births [4, 5]. Disrespectful and abusive treatment of women seeking obstetric care appears to be widespread and pervasive. According to the framework established by Bowser & Hill [6], it includes not only verbal and physical abuse, but also mistreatment such as discrimination, non-consented clinical care, denial of care, and detention in health facilities for failure to pay [7, 8]. These transgressions are important not only because they deter women from skilled delivery, but also because they are direct human rights violations that take place within the very medical establishments charged with helping women achieve “the right to the highest attainable standard of health” [9].

Disrespectful maternity care is not merely an interpersonal problem, but, rather, is driven by health system structures and social norms [10, 11]. Most system-level deficiencies lie outside of the control of individual providers. These include hospital overcrowding, inadequate resources, understaffing, and mistreatment of hospital workers themselves [12]. Such institutional factors promote provider burnout and non-empathic care. In addition, social inequalities tend to exist between patients receiving care in public hospitals and the providers caring for them. Providers’ deeply held, often unconscious biases based on race, class, and gender can lead to discrimination towards patients [11, 12].

The World Health Organization acknowledges that disrespectful care is “a powerful disincentive for women to seek and use maternal health care services.” [4] However, interventions to eliminate or mitigate abuse and mistreatment women in birthing facilities have to date been limited in both number and scope. Promising work in Tanzania [13] and Kenya [14] have implemented hospital staff training—including facilitated reflection on the motivators of unprofessional behavior of health workers—and shown improvements in respectful care.

In this article we respond to a recent call [15] for innovative approaches to promote respectful maternity care by presenting our model: obstetric care navigation. This approach trains lay women to facilitate referrals from home births to hospitals when complications arise, improving patient experience through accompaniment, translation, and labor support while simultaneously overcoming other barriers such as transportation. In this commentary, we explore the multifaceted role that care navigators play in facilitating higher quality respectful woman-centered care.

### Maternal heath in rural Guatemala

Recently, Guatemala was chosen as one of three global sites for an in-depth study of disrespectful and abusive maternity care [16]. This reflects the fact that this small Central American nation presents one of the most challenging landscapes for maternal health in the world. Although Guatemala’s overall maternal mortality rate (MMR) is 88 per 100,000, rates among indigenous Guatemalans, mostly of Maya descent, may be twice this high [1, 17].

Many factors contribute to this disparity. While indigenous Guatemalans represent at least 45% of the overall population [18], they control little of the country’s wealth and land rights [19]. A civil war that took place from 1960 to 1996 included state-sponsored killing of an estimated 300,000 Maya people [20]. Twenty years after the war, stark inequalities in healthcare for indigenous persons persist. Although free medical care, including prenatal care, is constitutionally guaranteed to all Guatemalan women through Ministry of Health facilities [21, 22], chronic underfunding, allegations of corruption, inadequate staffing, frequent medication stock-outs, and long wait times all contribute to low-quality care [23]. Furthermore, while about half of indigenous Guatemalans speak primarily a Mayan language [24], public services are available only in Spanish. Transportation also limits access to emergent facility-care, as most indigenous Guatemalans live in rural areas located far from hospitals [18].

In this context, over half of Maya women forgo institutional delivery and give birth at home with traditional midwives [18]. These “unskilled” attendants receive basic training from the Ministry of Health and integrate it with traditional practices [25]. When complications arise, traditional midwives are trained to refer patients to public facilities for obstetric care, but many women refuse or delay care [26]. Many especially fear hospital delivery, specifically citing unconsented sterilization as a factor [26]. In a recent study, three factors were strongly associated with a positive hospital birth experience: if the patient felt she was treated with respect by staff, if she was allowed accompaniment during labor, and if she was spoken to in her indigenous language [27]. While these rights are guaranteed by Guatemalan law, in practice they are rarely granted.

The concept of obstetric care navigation was born out of a collaboration between traditional midwives serving rural indigenous communities of the central Guatemalan highlands and Wuqu’ Kawoq | Maya Health Alliance (MHA), a non-governmental primary care organization in these same communities. This built on an earlier project to empower traditional midwives with better tools to detect preeclampsia in the community setting [28, 29]. In this project, detection at times did not translate into improved health outcomes because patients frequently refused to leave their communities for hospital care. It became clear to us that a new paradigm for facilitating referral care was needed.

### Care navigation: A brief history

Care navigators were first used to address the difficulties low-income minority patients in the United States faced in receiving timely cancer screening and treatment [30]. The original architects of care navigation programs witnessed how marginalized patients inordinately struggled to traverse the fragmented cancer care continuum [31]. Poor communication between patients and their doctors and barriers arising from limited economic resources, fear, and distrust exacerbated this challenge. In response, care navigators were trained to coordinate services (from community-based screening through cancer treatment) to help patients seamlessly flow through the disjointed health system. Care navigators develop one-on-one relationships with patients and provide motivation and emotional support, features that are shared with the patient accompaniment model popularized in global health for improving adherence to tuberculosis and HIV/AIDS treatment [32–35]. Care navigators have been shown to lessen delays in cancer diagnosis and treatment that contribute to poor outcomes and racial disparities [31, 36]. The model has since been adapted to improve chronic disease care, with some promising evidence of benefit in patients with limited English proficiency in the United States [37].

Over the past decade, MHA has developed a care navigation program to facilitate timely cancer care for Maya patients within Guatemala’s public hospitals [38]. Due to health system fragmentation and high out-of-pocket-costs, many poor or indigenous patients who present to the national cancer hospital do not complete treatment. For example, in a recent study of women with cervical cancer, only 35% of patients completed 5-year treatment plans due to loss-to-follow-up [39]. However, with the help of care navigators from MHA's complex care navigation program, many patients have successfully completed cancer treatment, and the program has now expanded to serve a wider variety of patients with chronic diseases, such as end-stage renal disease and congenital heart disease [38].

### Obstetric care navigation

**Fig. 1:**
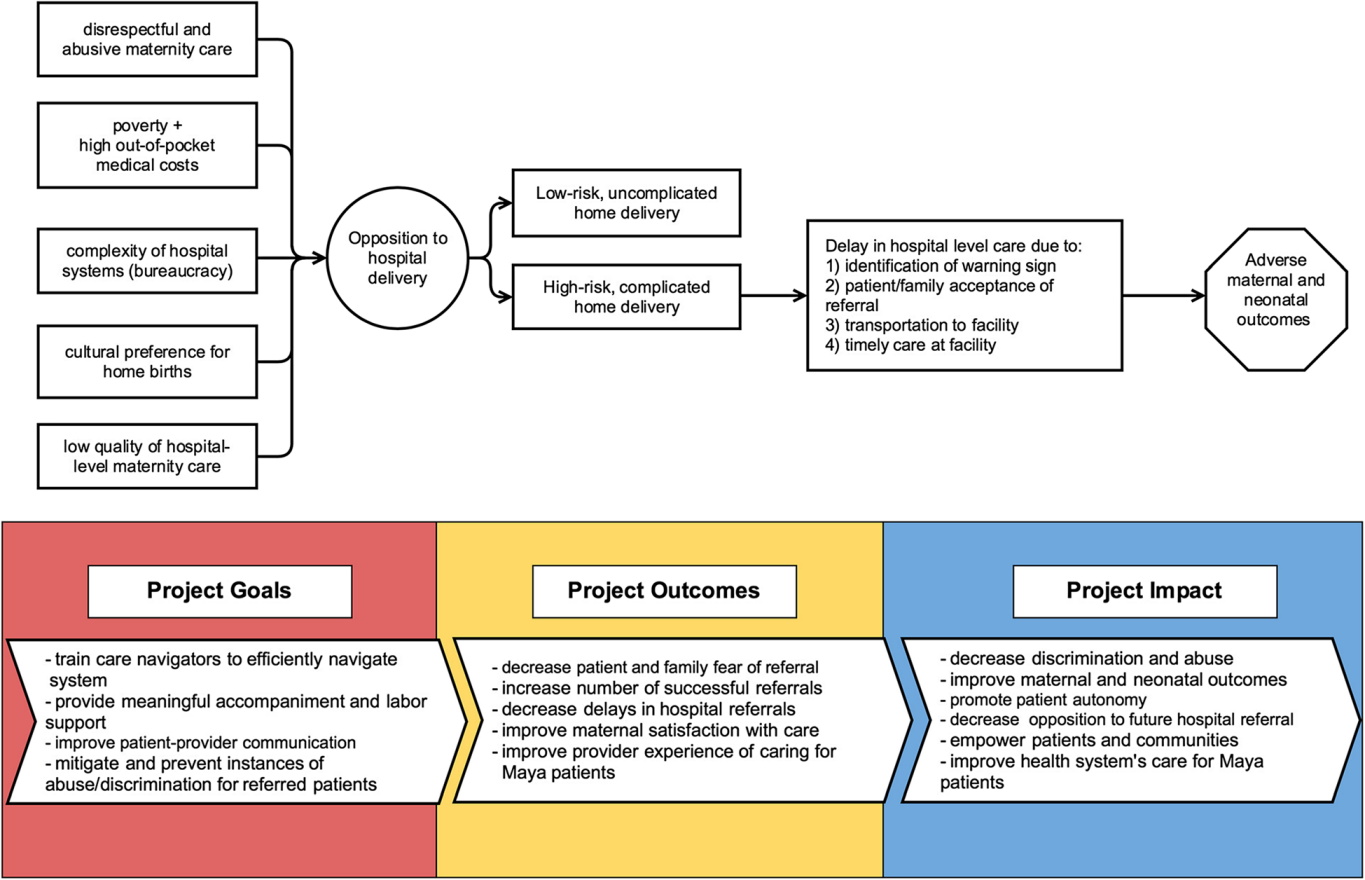
Theoretical model for obstetric care navigator program

Based on these preliminary experiences, we reasoned that a similar, carefully optimized care navigation model might also improve obstetric care. Therefore, in March 2017, we began a pilot program that employs care navigators to bridge the disjointed continuum of obstetric care from home delivery to hospital care. We devote the remainder of this commentary to describing the philosophy and design process for this program. We believe the approach may improve maternal and neonatal outcomes by preventing and mitigating disrespectful and abusive care in public hospitals and increasing rate of acceptance of medically-indicated referrals (Fig. 1). To our knowledge, no such program has ever been tested, though others have offered accompaniment at isolated points in the care continuum [40–42]. The pilot serves approximately 800 pregnant women/year from rural Maya communities in the central Guatemalan province of Chimaltenango. All women receive obstetric services from one of 45 collaborating traditional midwives, all of whom are credentialed through the Ministry of Health.

Participating midwives all are equipped with smart phones with a previously described decision support interface [28, 29] to communicate with the MHA clinical team when pregnancy and birth emergencies arise, ranging from the detection of preeclampsia during prenatal care to postpartum hemorrhage. The emergency line is staffed at all times by MHA health workers who—guided by simple clinical algorithms—support midwives’ decision making on whether to refer. When emergency referral is indicated, an on-call care navigator is notified. She then works with the midwife and patient’s family to arrange transport for the patient to the public hospital. If ambulance transport is not an option—a common occurrence in remote villages—a network of community members with vehicles is tapped to provide emergency transport.

Inside the hospital, care navigators provide a variety of services to improve quality of care and patient experience. Care navigators provide concrete assistance at all steps of the medical evaluation, such as translating between the Mayan languages spoken by most indigenous patients and Spanish-speaking nurses and physicians. As patients often find the hospital environment confusing and frightening, care navigators educate on expectations for the hospital experience, including information to explain or contextualize staff behaviors, brief clinical interactions, prolonged wait times, or visitation policies. They also provide updates for the patient’s family and traditional midwife. Obstetric care navigators are given small budgets to facilitate purchase of medications, supplies, or laboratory testing requested by the medical team but not available in public hospitals due to stocks outs. They can also use funds to purchase small comforts for the patient and her family, such as snacks or arrange for family accommodation.

During births, obstetric care navigators enter labor wards to assist women with position change (including traditional standing delivery), breathing techniques, and supportive coaching in Mayan languages. In the case of caesarean delivery, they are present in the operating room to offer emotional support. Once her patient is discharged or comfortably resting postpartum, obstetric care navigators return home. Depending on each patient’s individual needs care navigators may return to help facilitate hospital discharge, coordinate follow up, purchase medications, or even respond to a postpartum emergency that arises.

The pilot implementation team consists of a supervising doctor, nurse manager, field nurse, two community health workers, three care navigators, and collaborating midwives. Implementation is guided by the rapid-cycle quality improvement (RCQI) approach [43], including decisions about resource allocation and capacity for increasing volume of referrals. The leadership group includes the care navigators—drawn from the communities served by the project—and two elected midwives to represent these important perspectives. They meet every 2 weeks to review data on important performance indicators—such as referral volume and time to referral completion—in addition to qualitative feedback. This approach allows the team to quickly identify obstacles to referrals, empower frontline health workers to develop innovate solutions to implement on a small scale, and use data to assess effectiveness of these reforms. Care navigators’ compensation is tied to referral volume, including additional incentives for successful referrals. This is part of the larger effort to promote an outcomes-driven approach, to ensure efficient use of donor funds, and to titrate referral volume to budget capacity.

### Potential impact on disrespectful and abusive maternity care

We believe that obstetric care navigators hold the potential to improve the hospital experience for patients. Beyond the obvious practical services they provide, they also have the potential to address humanistic barriers to facility-based delivery by preventing disrespectful care through multiple mechanisms (Table 1). First, their presence as observers can deter mistreatment, enhanced by the collegial relationships they form with hospital staff over time. Second, their role as interpreters improves patient-provider communication and helps patients exercise autonomy, which includes ensuring that providers obtain informed consent before procedures. Third, they offer doula-like labor support that prevents the neglect and abandonment felt by many patients during hospital delivery. Importantly, doula services in the United States have been shown to reduce cesarean delivery rates [44], which raises an important future research question for our model of the navigators role as advocates. When disrespect and abuse does occur in medical care, care navigators offer emotional support and companionship to the affected woman. In addition, care navigators can perform small interventions to protect patient dignity, such as shielding a patient when she is must undress in an overcrowded hospital without privacy.

**Table 1:** Types of disrespectful and abusive care as categorized by Bohren and colleagues in their systematic review [5]. Each is paired with the systems-level drivers of this care (Propagating Factors) and specific supports that care navigators can provide in order to address them (Care Navigator Roles)

Type	Propagating Factors	Care Navigator Roles
1) Physical, sexual, or verbal abuse	Normalization of patient abuse Role modeling of behaviors by superiors	Mitigate through emotional support Deter through mediation
2) Discrimination	Social inequalities and segregation Lack of consequences and oversight for discriminatory treatment	Advocate for incorporation of traditional birthing practices Use cultural capital to identify both with patients and providers
3) Failure to provide professional standard of care (including patient’s autonomy)	Medical paternalism High patient volume Hierarchy of hospital staff Lack of mechanisms to measure and improve quality of care	Advocate for appropriate and timely care Assist providers in obtaining patient consent Take steps to protect patients’ dignity (ex: shielding while changing into gown in public exam room) Educate patients and hospital staff on mothers’ rights
4) Poor communication	Language barriers Lack of training on effective communication	Translate between patients and providers Report back to family and traditional midwife
5) Poor rapport	Social stratification Burnout-motivated behaviors	Act as cultural broker Provide emotional and labor support
7) Health system constraints	Provider frustration due to lack of resources Lack of basic resources to provide care Uncomfortable working conditions	Provide economic support for medical needs Contextualize limitations faced by medical providers for patients and family
8) Health system conditions	Excessive bureaucracy Inadequate support staff	Navigate complex work flows Coordinate care across settings (ex: between rural health post and hospital) Cultivate relationships with frontline health workers

The role of our obstetric care navigators, as a bridge between indigenous Maya communities and public hospitals, requires a unique skillset. On one hand, care navigators gain the trust and respect of midwives, patients, and their families, as they are themselves women from indigenous communities and native speakers of Maya Kaqchikel. To function in the medical setting and garner respect from non-indigenous staff, navigators must also be fluent in Spanish, facile with communication technology (including smart phones to document clinical encounters), and possess some formal education. Care navigators also develop strong communication skills, as they must deploy non-confrontational ways to advocate for their patients’ needs. We provide formal training in medical interpretation and how to facilitate informed consent discussions between patients and providers. Role-playing exercises help navigators gain motivational interviewing and conflict resolution skills. In addition, bimonthly team meetings also include time for reflection on difficult patient cases, and mental health resources are available for further staff support.

With respect to interactions with traditional midwives, our philosophy of care navigation springs in large part from direct, sustained collaboration with traditional midwives and their feedback as they usually do not feel comfortable accompanying patients to hospitals because they lack many of these aforementioned skills (especially Spanish language fluency and facility with technology), do not understand how to navigate the hospital, and often suffer discrimination. The care navigation model continues to support the role of traditional midwives as trusted health providers and important leaders in Maya communities [45], while providing additional complementary services at the referral level.

We hypothesize that a key feature of our obstetric care navigation model is that it also substantially benefits hospital-level providers by addressing the system deficiencies that fuel disrespectful and abusive care. For example, providers’ work becomes more efficient because Mayan language interpreters are on hand. Care navigators can also help overcome some of the frustrations of practicing medicine in a resource-poor facility, such as being able to enable requests for needed medications and laboratory tests that would otherwise go unmet. After hospitalizations, patients continue to receive care navigators’ support, making them more likely to adhere to treatment plans. Importantly, care navigators do not duplicate services already available within hospitals, but instead fill in gaps in care. They can also extend the capacity of positions that exist but are severely understaffed, such as a provincial referral hospital’s sole social worker.

To date, hospital staff have welcomed obstetric care navigators and the services they offer. Prior to implementation of our pilot, we involved heads of maternity services of the health centers and hospitals in Chimaltenango during planning stages and elicited their perspectives on barriers to care for indigenous women, drivers of disrespectful medical care, and specific non-clinical support obstetric care navigators could provide to their medical teams. Their suggestions led to numerous and ongoing program modifications, including use of colored uniforms to distinguish care navigators from providers of medical care in maternity areas. We also introduced care navigators to hospital staff prior to implementation in order to promote collegial relationships.

It is worth highlighting two ways in which our obstetric care navigation approach may differ from existing approaches to respectful maternity care [13, 14]. First, obstetric care navigation is a demand-driven approach that empowers communities to generate their own solutions to disrespectful obstetric care. Both care navigators and traditional midwives are drawn from the communities served by the program and have played an integral role in its conceptualization, and ongoing quality improvement (including participation in biweekly team meetings). While hospital-level interventions may complement the program we describe here, we believe obstetric care navigators are best able to gain trust and adapt to local needs, in no large part because they operate in a complementary fashion at the community level and outside Ministry of Health governance and human resources models.

### Uniting respectful maternal care and quality of care

Disrespectful and abusive care is inseparable from larger concerns about the low quality of maternity care in LMICs. Women and their families have long raised these concerns about public health facilities, which cannot be explained solely by communication barriers and cultural misperceptions. Promoting facility-based delivery without concurrent efforts to improve quality may paradoxically increase maternal mortality. Indeed, within some areas of Guatemala rates of maternal death are higher in hospital as compared to home births with traditional midwives. Simply recommending universal hospital delivery would cause further strain and likely decrease quality further. By guaranteeing emergency services for women who opt for home births, the care navigator model selects only those with complications for facility-based deliveries and allocates scarce hospital resources to the women who face the highest risk of maternal mortality. As such, services can be focally deployed in communities with the highest maternal mortality rates to determine the greatest impact for cost.

Our pilot program includes components explicitly directed at improving quality of care beyond just the obvious interpersonal and communication roles played by the navigator. First, care coordination efforts seek to overcome the disjointed referral chain and reduce loss-to-follow-up. Transportation is provided not only for emergency referrals, but also for outpatient visits to facilitate earlier detection and appropriate care for high-risk pregnancies and for postpartum follow up. Second, the lead physician is in frequent communication with providers in public facilities of the catchment area, working to overcome the limits of existing referral mechanisms. In addition, this physician audits care patients receive and communicates treatment recommendations to public sector physicians. Finally, quality improvement efforts in low-resource settings are often limited by inadequate data collection and analysis infrastructure. However, in our pilot project, all community-level data generated are shared with public facilities to help them better understand out-of-hospital needs and longitudinal patient outcomes. This process is greatly facilitated by the project’s use of a centralized electronic medical record.

## Conclusions

While obstetric care navigation alone will not solve the problem of disrespectful and abusive obstetric care, care navigators offer a significant incremental improvement in the experience of maternity care. The collective impact of their interactions with patients and providers has the potential to be transformative. Profound cultural and contextual differences between indigenous patients and non-indigenous providers enable disrespectful behavior on the part of providers and leads to unrealistic patient expectations. Obstetric care navigators facilitate a shared understanding that humanizes patients to providers and vice versa. Care navigators’ presence can also play an important role in changing institutional culture, especially when they model labor support techniques and provide passive education on informed consent. In time, these forces may help to break the self-perpetuating cycle of disrespectful and abusive care, leading to improved utilization, patient satisfaction, and maternal outcomes, as well as provider engagement in caring for indigenous populations.

Currently, we are in the process of collecting outcomes data on the objective success of our pilot, which will conclude in April 2018. This includes careful assessment of improvements in obstetrical referral success rates, referral times, and adherence to medical treatment. We are also closely tracking patient satisfaction and experience metrics along with qualitative assessment of the program’s potential impact. Following completion of this demonstration project we hope to share our results and partner with other communities, NGOs, and governments to disseminate the model. Rigorous implementation science methods will be needed to adapt the model to local needs, engage communities and stakeholders, and monitor success. We invite implementers and funders alike to join us and others in investigating the role obstetric care navigation and other patient accompaniment models can play in the development of community-driven solutions which promote respectful maternity care.

## Abbreviations

LMIC: low and middle-income countries
MHA: Maya Health Alliance
